# Virus-Negative Necrotizing Coronary Vasculitis with Aneurysm Formation in Human SARS-CoV-2 Infection

**DOI:** 10.3390/idr13030055

**Published:** 2021-06-24

**Authors:** Andrea Frustaci, Marco Francone, Romina Verardo, Maria Rosaria Capobianchi, Cristina Chimenti

**Affiliations:** 1 Department of Clinical, Internal, Anesthesiologist and Cardiovascular Sciences, Sapienza University, 00166 Rome, Italy; 2 Department of Radiological, Oncological and Pathological Sciences, Sapienza University, 00166 Rome, Italy; marco.francone@hunimed.eu; 3 Cellular and Molecular Cardiology Lab, IRCCS L. Spallanzani, 00149 Rome, Italy; romina.verardo@inmi.it; 4 Laboratory of Virology National Institute for Infectious Diseases “L. Spallanzani”, 00149 Rome, Italy; maria.capobianchi@inmi.it (M.R.C.); cristina.chimenti@uniroma1.it (C.C.)

**Keywords:** necrotizing coronary vasculitis, SARS-CoV-2, myopericarditis

## Abstract

We report a case of myopericarditis associated to SARS-CoV-2 infection with necrotizing coronary vasculitis of intramural vessels, giving rise to biventricular apical microaneurysms and to electrical instability. Negativity of myocardial polymerase chain reaction for the most common cardiotropic viruses and for SARS-CoV-2 suggested an immune-mediated myocardial and pericardial inflammatory disease. High dose (1 mg/Kg daily) prednisone and anti-viral (Remdesivir, IDA Business, Carrigtohill, County Cork, T45 DP77, Ireland) therapy led to resolution of cardiac inflammation and ventricular arrhythmias. Morpho-molecular characterization of endomyocardial tissue may improve the outcome in subjects with SARS-CoV-2-associated myopericarditis and coronary vasculitis.

## 1. Introduction

Cardiac inflammation may occur during SARS-CoV-2 infection and even contribute to death of affected subjects [1]. Involvement of myocardium and pericardium is mostly recorded while little is known on epicardial and intramural coronary vessels. Identification and treatment of coronary vasculitis is still unreported. We reported the case of a women with SARS-CoV-2 infection, who developed a myocarditis associated with vasculitis of intramural coronary vessels responsive to immunosuppressive treatment.

## 2. Methods and Results

A 50-year-old female was admitted because of fever, dry cough and shortness of breath. Her past clinical history was silent. Polymerase chain reaction testing a nasopharyngeal swab was positive for SARS-CoV-2. Chest computed tomographic imaging showed severe pneumonitis with multiple ground-glass opacities with a confluent appearance mainly at the level of the lower lobes, the lateral segment of the middle lobe and the lower and posterior lingular segments of the right upper lobe.

**Figure 1:**
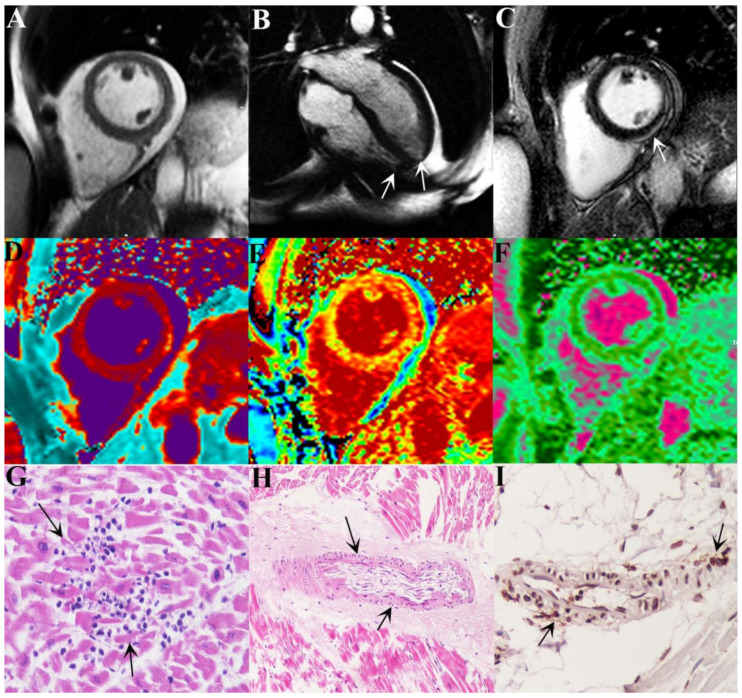
Virus-negative Necrotizing Coronary Vasculitis with Aneurysm Formation in Human Sars-Cov-2 Infection; (**A**,**B**) Cardiac magnetic resonance (CMR) shows normal LV dimensions and function (LV ejection fraction of 59%) (**A**) and the presence of right and left apical microaneurysms (see arrows in **B**); (**C**,**D**) Late gadolinium enhancement (LGE) imaging showing a subepicardial area in the infero-lateral wall on basal and mid-ventricular planes (**C**); corresponding mapping sequences documents a focal increase in native T1 values (**D**); (**E**,**F**) Extracellular volume fraction (**E**) and T2 mapping values (**F**) are consistent with diffuse edema and combined extracellular matrix expansion; (**G**) Left ventricular endomyocardial biopsy reveals diffuse lymphomononuclear infiltrates associated with necrosis of adjacent myocytes consisting with myocarditis. (EE magnification, 200×); (**H**,**I**) show necrotizing vasculitis of intramural coronary arteries with strong positivity for CD45RO+ T-lymphocytes. (EE and IHC, magnification 200×).

**Figure 2:**
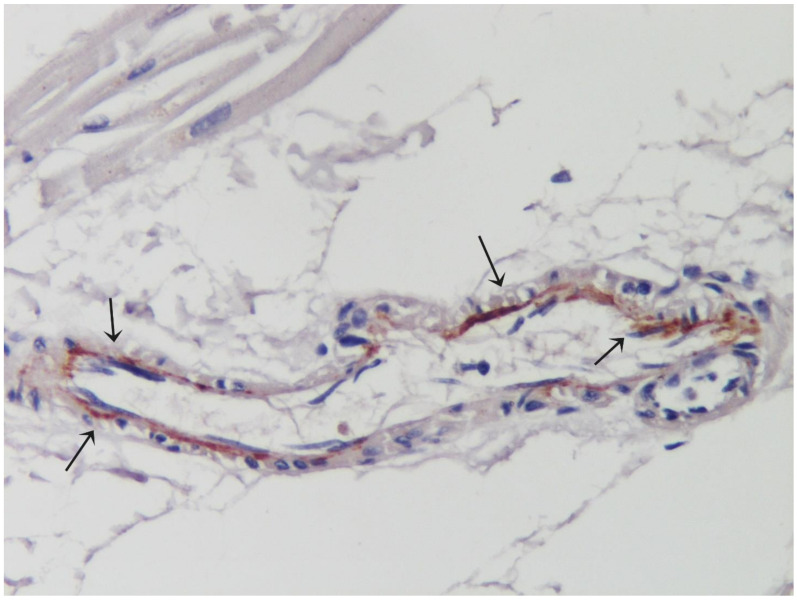
IHC for C3d shows deposition of complement fraction C3d. Deposition of complement fraction C3d in the endocardium of an arteriole suggesting endothelitis. (IHC 200×).

During hospitalization, she became symptomatic for angina and palpitations. Electrocardiogram showed sinus rhythm (75 bpm) with diffuse and non-specific repolarization abnormalities, QT interval was within limits (460 ms). Holter monitoring showed frequent ectopic ventricular beats and several sequences of non-sustained ventricular tachycardia. Routine laboratory tests revealed elevation of cardiac troponin HS (0.077 mcg/L, nv < 0.014 mcg/L), C-reactive protein (1.7 mg/dL, nv 0.00–0.50 mg/dL) and mild lymphocytopenia (0.8 × 109/L, nv 1.00-4.80 × 109/L). All the other blood tests, including serum levels of C3 and C4, were within normal limits. Two-dimensional echocardiography showed normal size and function of the left ventricle (LV) with mild pericardial effusion. Cardiac magnetic resonance (CMR) confirmed normal LV dimensions and function (LV ejection fraction of 59%) (Figure 1A) and revealed the presence of right and left apical microaneurysms (Figure 1B and Supplementary Materials). Late gadolinium enhancement (LGE) imaging showed a subepicardial area in the infero-lateral wall on basal and mid-ventricular planes (Figure 1C); corresponding mapping sequences documented a focal increase in native T1 values (Figure 1D), extracellular volume fraction (Figure 1E) and T2 mapping values (Figure 1F) consistent with diffuse edema and combined extracellular matrix expansion. These CMR features were highly suggestive for acute myo-pericarditis. In order to investigate a possible involvement of coronary circulation and the mechanism, virus-induced or immune-mediated, operating, the patient underwent an invasive cardiac study including coronary angiography and LV endomyocardial biopsy (EMB) after written informed consent. Epicardial coronary arteries were normal; endomyocardial biopsy showed diffusely mononuclear infiltrates associated with necrosis of adjacent myocytes (Figure 1G) and necrotizing vasculitis of intramural coronary arteries (Figure 1H) associated to positivity of CD45RO+ T-lymphocytes (Figure 1I) of the affected vessel wall. Arterioles not infiltrated by inflammatory cells presented at immunohistochemistry complement fractions (C3d) deposition (Figure 2) suggesting coexisting endothelitis. Moreover, real time PCR on two frozen endomyocardial samples for SARS-COV-2, and for the most common cardiotropic viruses including adenovirus, cytomegalovirus, parvovirus B19, Epstein–Barr virus, human herpes virus 6, and herpes simplex virus 1 and 2, enterovirus, influenza virus A H1N1 and B and hepatitis C virus was negative. These findings were consistent with immune-mediated myocarditis with necrotizing coronary vasculitis and aneurysm formation in SARS-COV-2 infection. Treatment included ramipril, bisoprolol and prednisone (1 mg/Kg daily) in addition to anti-viral therapy with remdesivir. At 1 month follow-up C-reactive protein and troponin HS values normalized, pericardial effusion resolved at echocardiogram while cardiac contractility remained preserved. Lung CT was normal and nasopharyngeal swab negative for SARS-COV-2. Control Holter monitoring failed to record ventricular arrhythmias.

## 3. Discussion

The present report shows as necrotizing coronary vasculitis of intramural vessels may associate to myopericarditis in human coronavirus infection. It may give rise to electrical instability and myocardial deterioration contributing to death of SARS-COV-2 affected subjects. The occurrence of myocardial inflammation has been described in some patients with SARS-COV-2 [1], but the involvement of intramural vessels has never been described before. Myocardial PCR for SARS-COV-2 and most common cardiotropic viruses was negative suggesting an immune-mediated mechanism of damage. To this regard, systemic endothelitis [2] with deposition of complement fractions has been documented. Our report suggests (Figure 2) that even coronary microcirculation can be as well a site of immunocomplexes deposition and immune-mediated damage. This mechanism is potentially susceptible to immune-modulating therapy as inhibitors of interleukin 1B, interleukin 6 and steroids in combination to anti-viral therapy [3,4]. The intramural small vessels vasculitis detected in our patient is different from classical Kawasaki disease, that involves medium sized arteries with aneurysm formation and occurs mainly in children <5 years old. However, SARS-COV-2 has been implicated in a Kawasaki-like disease in children with COVID-19 associated inflammatory multisystemic syndrome [5]. Intramural coronary vasculitis in patients affected by SARS-COV-2 infection can be suspected by presence of myocardial microaneurysms at CMR. Indication to ventricular endomyocardial biopsy may rise for the occurrence of mechanical deterioration or electrical instability questioning on the possible use of immunosuppression or high dose steroids. Introduction of this therapy, however, requires excluding by myocardial PCR, persistence of SARS-COV-2 or presence of additional cardiotropic viral agents [6]. EMB as investigation of this entity has been already extensively applied with no complications as in our case. Use of steroids in the treatment of SARS-COV-2 has been firstly discouraged by World Health organization then it has been recommended following extensive trials [7,8] reporting reduction of mortality up to 35% of SARS-COV-2 patients. Identification of virus-negative intramural coronary vasculitis definitely suggests adoption of high dose steroids. 

In our report this last treatment was well tolerated and allowed the cure of pericardial effusion as well as control of ventricular arrhythmias.

## Data Availability

Not applicable.

## Supplementary Materials

The following are available online at https://www.mdpi.com/article/10.3390/idr13030055/s1.
